# Urban-rural differences in disability-free life expectancy in Bangladesh using the 2010 HIES data

**DOI:** 10.1371/journal.pone.0179987

**Published:** 2017-07-24

**Authors:** Md. Shariful Islam, Md. Ismail Tareque, Md. Nazrul Islam Mondal, Ahbab Mohammad Fazle Rabbi, Hafiz T. A. Khan, Sharifa Begum

**Affiliations:** 1 Department of Population Science and Human Resource Development, University of Rajshahi, Rajshahi, Bangladesh; 2 Department of Statistical Sciences, University of Padua, Padua, Italy; 3 Graduate School, The University of West London, St Mary’s Road, Ealing, London, United Kingdom; 4 Population Studies Division, Bangladesh Institute of Development Studies (BIDS), Dhaka, Bangladesh; Yale University Yale School of Public Health, UNITED STATES

## Abstract

**Background:**

Research on health expectancy has been carried out in Bangladesh but none of it has examined the differences in Disability-Free Life Expectancy (DFLE) between urban and rural setting in context of rapid urbanization of Bangladesh in past decades.

**Objectives:**

The present study aims to estimate DFLE for people of all ages living in urban and rural areas in Bangladesh, and to examine the differences in DFLE between these two areas.

**Methods:**

Data from the Sample Vital Registration System 2010 and the Bangladesh Household Income and Expenditure Survey (HIES) 2010 were used in this study. The Sullivan method was applied to estimate DFLE in Bangladesh.

**Results:**

Higher rates of mortality and disability were observed in rural areas compared to urban areas with few exceptions. Statistically significant differences in DFLE were revealed from birth to age 15 years for both sexes between urban and rural areas. Urban males had a longer life expectancy (LE), longer DFLE and shorter LE with disability both in number and proportion when compared to rural males. Rural females at age 20+ years had a longer LE than urban females but urban females had a longer DFLE and a shorter LE with disability in both number and proportion at all ages than did rural females.

**Conclusion:**

This study demonstrates that there were clear inequalities in LE, DFLE and LE with disability between rural and urban areas of Bangladesh along with age-specific differences as well. These findings may serve as useful and benchmark for intervention and policy implications for reducing the gap in health outcomes.

## Introduction

Until the late 19^th^ century, life expectancy (LE) had been seen as a sufficient indicator for determining population health and public health priorities [1]. People across the world now expect to have longer lifespans and longer disability-free life expectancy (DFLE). DFLE or healthy life expectancy has gained momentum over LE for assessing population health and public health priorities around the world, particularly in developed countries. DFLE focuses on the quality of life whereas LE measures the quantity of life a person expects to live. It is a valuable index for understanding changes in both the physical and mental health states of the general population, for allocating resources, and for measuring the success of political programmes [2, 3]. Several measures of health state, such as self-rated health, cognitive impairment, activity limitation, functional limitation, work-loss day, and disability had been used in the computation of health expectancy in earlier studies. Although a remarkable heterogeneity in the choice of health indicators was discovered in the computation of health expectancy [4], health expectancy was also reported to be a useful and convenient measure for monitoring and assessing quality of life and for comparing different groups and populations [5].

A large number of studies on health expectancy have been carried out in developed regions [6–9], but a relatively lower number of studies, particularly for older adults, were found to have been carried out in developing regions and in Asian countries [10–12]. Between 1986 and 2004, morbidity compression until 1995 followed by an expansion of morbidity were reported for Japanese population [13]. Among older Thais, the average number of years living with and without morbidity and disability as measured by multiple dimensions of health (e.g. chronic disease; cognitive impairment; depression; disability in instrumental activities of daily living; and disability in activities of daily living) were varied including inconsistent gender differences across the measures [14]. In China, wide regional variations were reported in DFLE at age 60 with per capita gross domestic product, proportion of urban residents, and access to health care being the primary factors associated with these geographical variations [12]. Among China, Ghana, India, Mexico, Russia and South Africa it was China that had the highest DFLE and India that had the lowest DFLE. In each country, women lived longer than men, but women had more disabilities in both absolute and proportional terms [15].

Bangladesh, a country of South Asia, has a population of 158.90 million with a density of 1077 people per square km [16] and is ranked as the world’s eighth and Asian’s fifth most populous country. In Bangladesh, several studies have been conducted on healthy expectancy with particular emphasis on the older population. Despite having longer LE, women aged 60+ years had a greater prevalence of disability and shorter DFLE than men of age 60+ years over the whole country [17]. Healthy life expectancy declined significantly with increases in age among older adults of 60+ years in the Rajshahi district of Bangladesh [18]. Individuals at age 60 expected about 41% of their remaining life to be in good health, while individuals at age 80 expected only 21% of their remaining life to be in good health. Exercise, sufficient income, physical limitations and facing abusive behavior were reported to be modifiable to a certain extent in order to provide the potential for improving healthy life expectancy in the Rajshahi district [19]. A study found improvements in the mean number of work loss days as well as improvements in partial work loss free life expectancy among the male population aged 15–54 years between 2004 to 2007 [20]. Among the people aged 15+ years, men were found to expect fewer life years in numbers to be spent in good health with a much larger proportion in good health compared to women during 1996–2002 [21]. There has however been no study found on health expectancy that covers all ages of Bangladeshi population.

Rapid urbanisation has been observed in Bangladesh over the last decades with around 76.9% of the total population living in rural areas and the rest living in urban areas [22]. People living in urban areas were better off in terms of housing, education, income, health facilities, and sanitation than people living in rural areas [23]. When compared with rural areas, people living in urban areas were reported to have a higher rate of hypertension [24] but a lower rate of disability [25]. However, DFLE at birth and at different ages within the urban-rural differentials have never been studied in Bangladesh. To fill the gap, we examined the differences in DFLE in terms of place of residence.

## Methods

Two key pieces of information, namely (i) mortality information (age-sex specific mortality rate) and (ii) morbidity information (disability prevalence) were used from two large nationally representative sample surveys in order to compute DFLE. At first, age-sex specific mortality rates by urban and rural areas were obtained from the Sample Vital Registration System 2010 [26] to generate sex-specific period life tables for urban and rural areas. Then, age-sex specific disability prevalence by urban and rural areas, obtained from the Bangladesh’s Household Income and Expenditure Survey (HIES) 2010 [27] were combined with period life tables to compute DFLE for urban and rural areas. All estimations were performed separately for males and females, and for urban and rural areas. The disability prevalence was estimated without applying sampling weights since no sampling weights were provided by HIES 2010.

### Estimation of period life tables

Conventional life table notations are used throughout this study. Observed mortality rate, _*n*_*m*_*x*_, from the Sample Vital Registration System 2010 were converted into the probabilities of dying, _*n*_*q*_*x*_. The Bangladesh Bureau of Statistics (BBS), the only national statistical institution that is responsible for collecting, compiling, and disseminating statistical data on vital statistics and population related matters for Bangladesh, released the Sample Vital Registration System 2010. The probabilities of dying were calculated using the following formula:

$${}_{n}q{}_{x}=\frac{n.{}_{n}m{}_{x}}{1+(n-{}_{n}a{}_{x}){}_{n}m{}_{x}}.$$

Where, *n* refers to the length of the age group and _*n*_*a*_*x*_ refers to the average number of person-years lived in the interval *x* to *x* + *n* by those dying in the interval.

As _*n*_*a*_*x*_ for Bangladesh in 2010 was unavailable, the Swedish 1945’s _*n*_*a*_*x*_ taken from the Human Mortality Database [28] were used for the conversion from _*n*_*m*_*x*_ to _*n*_*q*_*x*_. According to the methods of choosing _*n*_*a*_*x*_ [29] the Swedish 1945’s _*n*_*a*_*x*_ were found suitable for calculating period life tables for Bangladesh in 2010. The set of Swedish 1945’s _*n*_*a*_*x*_ from the male life table were used in the computation of both urban male and rural male life tables for Bangladesh in 2010, while the set of Swedish 1945’s _*n*_*a*_*x*_ from the female life table were used in the computation of both urban female and rural female life tables for Bangladesh in 2010. Finally, following usual procedures for period life table calculations as described by Preston and others [29] and using the set of calculated _*n*_*q*_*x*_, sex-specific period life tables were computed for Bangladesh in 2010 for urban and rural areas.

### Estimation of disability prevalence

The age-sex specific disability prevalence by urban and rural areas was estimated from the HIES 2010. The HIES 2010 was produced by the BBS and provided data on household income and expenditure as well as individual-level data on education, employment, health and disability. Based on a two-stage stratified random sampling technique, a total of 12,240 residential households were selected and of these, 4,400 households were from urban areas and 7,840 were from rural areas. From the selected households, a total of 55,580 males and females from the age of 5 years and above were interviewed. The data collection was carried out between 1 February 2010 and 31 January 2011. During data collection period, interviewers entered each household’s information into their laptops at the end of each day. They went back to the corresponding households if they found any inconsistencies or missing and made the required changes to the data [27]. Consequently, for the variables we used, there are no missing values in the dataset. The HIES 2010 did not collect disability data from children below 2–3 years due to absence of necessary cognitive performance. The sample was therefore restricted to those who were between the ages of 5 and 110 years old that provided a final sample of 49,885 individuals, of which 17,854 (35.79%) were from urban areas and the remainder from rural areas.

The HIES 2010 adopted and accommodated the short set of six disability-related questions endorsed by the Washington Group in the very long questionnaire for household income, household expenditure, individual education, employment, health etc. The short set of six disability-related questions was consistent with the International Classification of Functioning, Disability and Health, a framework for conceptualising disability developed by the World Health Organisation [30]. The six questions were:

1. Do you have difficulty seeing, even if wearing glasses?
2. Do you have difficulty hearing, even if wearing a hearing aid?
3. Do you have difficulty walking or climbing steps?
4. Do you have difficulty remembering or concentrating?
5. Do you have difficulty with self-care such as washing all over or dressing, feeding, toileting, etc.? and,
6. Do you have difficulty communicating, for example, understanding or being understood?

Each question had four response categories: (a) no difficulty, (b) yes, some difficulty, (c) yes, severe difficulty, or (d) yes, cannot perform at all. We have defined disability in two ways. Firstly, an individual was considered having disability if s/he were (1) having severe difficulty with any of the six indicators; or (2) being unable to perform any of the six indicators at all; or (3) having some difficulty with at least two of the six indicators. This approach to measuring disability had been used in a study for Uganda [31], and a similar approach had been used in several other studies [32, 33]. Secondly, an individual was considered disabled if s/he were having some difficulty with at least any of the six indicators. This approach to measuring disability had already been used in studies for Bangladesh [17, 25]. Disability prevalence was found higher in the second approach compared to the first approach.

### Estimation of disability-free life expectancy

The estimated life tables and disability prevalence from the above two approaches were combined using the Sullivan method [34] to compute DFLE in the current study. In accordance with the Sullivan method, the DFLE at age *x* (*DFLE*_*x*_) was calculated by using the following formula:

$$DFLE_{x}=\frac{1}{l_{x}}\sum_{a=x}^{\omega }L_{a}\pi_{a}.$$

Where *l*_*x*_ refers to the number of survivors at age x; *L*_*a*_ refers to the person-years lived for the age interval *a*, and *π*_*a*_ refers to the prevalence of disability-free for the age interval *a*. Details of the estimation procedure of the Sullivan method for calculating DFLE with confidence intervals are available elsewhere [35].

Using the two rates of disability prevalence, we estimated two DFLE for all ages, both sex, and urban-rural areas. Relatively higher differences in DFLE between urban and rural areas were found when we used disability prevalence from the second approach than that from the first approach. As we found similar levels of significance for the differences in DFLE in both approaches, and the second approach had been used in previous studies for Bangladesh, in the present study we reported the results that used second approach to measuring disability. All results can however be obtained from the corresponding author.

### Ethical considerations

The Ethics committee at BBS approved a waiver from ethical approval for this retrospective study. As the de-identified data for this study came from secondary sources, this study did not require ethical approval.

## Results

### Urban-rural differences in age-sex-specific mortality rate and disability prevalence

Table 1 shows the sex-specific mortality rate and disability prevalence for urban and rural areas for all ages-groups in Bangladesh in 2010. Age standardized mortality rate was the highest (7.75 per 1000) for the rural male population while the age standardized disability prevalence was the highest (9.83%) for the rural female population. The mortality rates among the oldest age groups (80+ years) were the highest in both urban and rural areas. The mortality rate for females was higher in rural areas than in urban areas across all groups except for age 55+ years, the same was true for males with a few exceptions in the 10–20, 35–40 and 70–75 year-old age groups. Steep rises in mortality rates from the age of 40 were observed for both areas and sexes, with the highest mortality rate for rural males.

**Table 1:** Age-sex-specific mortality rate (per thousand) and disability prevalence (per cent) for urban and rural areas in Bangladesh, 2010.

Age (years)	Age-sex-specific mortality rate (per thousand)				Age-sex-specific disability prevalence (per cent)					
	Male		Female		Male			Female		
	Urban	Rural	Urban	Rural	Urban	Rural	p-values^b^	Urban	Rural	p-values^b^
0^a^	31.51	45.12	32.73	35.05	1.56	2.34	0.14	1.30	1.57	0.55
1^a^	1.59	2.73	1.57	2.33	1.56	2.34	0.14	1.30	1.57	0.55
5	0.57	0.98	0.68	1.18	1.56	2.34	0.14	1.30	1.57	0.55
10	1.47	1.23	0.82	0.96	3.30	2.96	0.58	2.83	2.11	0.20
15	1.06	0.98	0.67	1.21	2.53	3.14	0.36	1.91	2.93	0.11
20	1.52	0.95	0.98	1.53	2.18	3.04	0.24	2.20	2.52	0.60
25	1.14	1.84	0.94	1.57	2.24	3.28	0.18	3.56	5.34	0.04
30	0.90	2.03	0.63	1.12	4.42	4.81	0.71	7.66	7.00	0.59
35	2.52	1.65	1.63	1.86	5.09	8.07	0.02	10.96	10.99	0.98
40	3.36	2.73	2.79	2.80	9.14	11.29	0.18	15.68	15.70	0.99
45	5.14	5.21	4.25	3.98	10.89	14.65	0.04	20.12	19.87	0.91
50	8.63	10.67	7.99	10.61	16.18	17.97	0.43	26.38	27.97	0.59
55	13.58	14.14	16.05	11.66	22.58	22.50	0.98	26.45	28.00	0.64
60	22.00	25.04	20.55	21.68	26.80	29.18	0.50	32.37	35.77	0.39
65	34.64	38.54	31.39	30.81	28.24	34.21	0.17	37.84	41.72	0.43
70	55.28	49.55	50.18	45.36	37.10	47.06	0.06	44.44	55.60	0.04
75	99.27	84.31	101.18	81.75	52.78	49.32	0.63	58.33	61.11	0.70
80+	135.22	143.22	128.26	127.48	60.00	64.11	0.55	68.13	66.18	0.74
Total*	**5.56**	**7.75**	**4.86**	**6.34**	**6.54**	**8.70**	**0.00**	**8.24**	**9.83**	**0.00**

^a^ As HIES-2010 did not collect disability information for ages 0–4, the disability prevalence at age 5 were used as disability prevalence at age 0 and 1

^b^ p values are for percent difference of disability prevalence between urban and rural areas

* age-standardized mortality rate and disability prevalence were calculated by direct method with the use of Bangladesh Population Census 2011 data.

The disability prevalence increased with age, particularly after 40 years. Rural males and females had respectively higher disability rates than urban males and females across almost all ages. However, only statistically significant differences between urban and rural areas were found for males at age 35 and 45 and for females at age 25 and 70.

### Urban-rural differences in LE and DFLE

**Fig 1:**
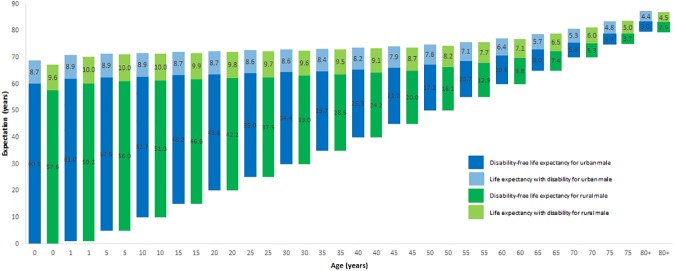
Urban-rural differences in DFLE and LE with disability for Bangladeshi male in 2010.

Table 2 shows LE, DFLE with a 95% confidence interval, proportion of expected life without disability for urban and rural areas, and the urban-rural differences in DFLE for all age-groups for the Bangladeshi male population in 2010. Urban males could expect to live longer than rural males across all ages except for ages 65–75 years. The life expectancy at birth for urban males was 68.77 years which was 1.56 years longer than for rural males. Out of 68.77 years, an urban male at birth could expect to live 60.12 years without disability (i.e. 87% of life years as disability-free) and 8.65 years with disability (see Fig 1).

**Table 2:** Urban-rural differences in life expectancy, disability-free life expectancy, and proportion of expected life without disability for Bangladeshi male in 2010.

	Life expectancy			Urban		Rural		Proportion of life without disability			
Age	Urban	Rural	Differences in LE^a^ (Urban—Rural)	DFLE	95% CI	DFLE	95% CI	Urban	Rural	Differences in DFLE^b^ (Urban—Rural)	p value^c^
0	68.77	67.21	1.56	60.12	59.54–60.70	57.60	57.19–58.02	87.42	85.70	2.52	0.00
1	69.94	69.26	0.69	61.03	60.43–61.64	59.23	58.80–59.67	87.26	85.53	1.80	0.00
5	66.38	66.00	0.38	57.48	56.87–58.08	55.96	55.52–56.40	86.59	84.79	1.52	0.01
10	61.56	61.31	0.25	52.71	52.10–53.32	51.34	50.90–51.78	85.62	83.74	1.37	0.02
15	57.00	56.67	0.32	48.25	47.64–48.86	46.79	46.35–47.23	84.65	82.56	1.46	0.01
20	52.28	51.94	0.35	43.61	43.00–44.22	42.16	41.72–42.60	83.42	81.18	1.45	0.01
25	47.66	47.17	0.49	39.04	38.43–39.65	37.50	37.06–37.94	81.90	79.50	1.53	0.01
30	42.92	42.59	0.34	34.36	33.75–34.97	32.99	32.55–33.44	80.05	77.47	1.37	0.02
35	38.11	38.00	0.11	29.73	29.11–30.34	28.55	28.10–28.99	78.01	75.13	1.18	0.05
40	33.56	33.29	0.27	25.33	24.71–25.94	24.17	23.73–24.60	75.47	72.59	1.16	0.05
45	29.08	28.71	0.37	21.17	20.56–21.79	20.03	19.60–20.46	72.80	69.77	1.14	0.05
50	24.77	24.40	0.37	17.21	16.59–17.82	16.23	15.81–16.66	69.47	66.53	0.97	0.10
55	20.75	20.60	0.15	13.68	13.06–14.30	12.90	12.48–13.33	65.93	62.65	0.77	0.20
60	17.02	16.91	0.11	10.62	10.00–11.25	9.82	9.40–10.25	62.42	58.09	0.80	0.20
65	13.70	13.83	-0.13	7.98	7.35–8.61	7.35	6.92–7.78	58.25	53.17	0.63	>0.20
70	10.81	11.23	-0.42	5.55	4.90–6.21	5.27	4.83–5.71	51.38	46.91	0.29	>0.20
75	8.46	8.68	-0.22	3.67	2.97–4.38	3.73	3.28–4.17	43.42	42.93	-0.05	>0.20
80+	7.40	6.98	0.41	2.96	2.08–3.84	2.51	2.05–2.96	40.00	35.89	0.45	>0.20

DFLE = disability-free life expectancy; CI = confidence interval of DFLE

^a^ differences are not statistically tested due to lack of required information

^b^ differences are statistically tested with z-statistic

^c^ p values are level of significance for a two-tailed test for differences in DFLE between urban and rural areas.

At almost all ages, urban males could expect to have longer LE, longer DFLE and shorter LE with disability than rural males (Fig 1). Life expectancy at birth was 1.56 years higher in the case of urban males compared to rural males, whereas DFLE at birth was 2.52 years higher in the case of urban males than rural males. Statistically significant differences in DFLE between urban and rural areas across all ages except for 50+ years were found for male population. In terms of numbers, the differences in DFLE between urban and rural areas were higher than the differences in LE at every age. The differences in LE and in DFLE between urban and rural areas were lower in higher age groups. At each age, an urban male expected a higher proportion of life without disability than a rural male. An urban male at birth could expect 87% of their life years to be without disability, and at age 65, an urban male could expect 58% of their remaining life years to be without disability. But a rural male at birth could expect 86% of their life years to be without disability and 53% of their remaining life years to be without disability at age 65. At open ended interval (80+ years), an urban and rural male, respectively, could expect only 40% and 36% of their life years to be without disability.

**Fig 2:**
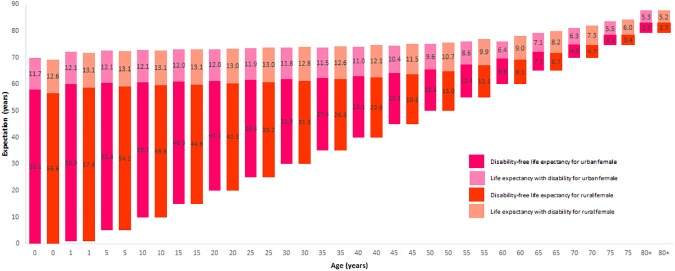
Urban-rural differences in DFLE and LE with disability for Bangladeshi female in 2010.

Table 3 and Fig 2 show LE, DFLE, and the urban-rural differences in DFLE for all age-groups for the Bangladeshi female population in 2010. Urban females could expect to live longer than rural females at age 0 to 10, but at age 20+ years urban females could expect shorter lives than rural females. However, urban females were found to expect longer DFLE than rural females except for age groups 75 and 80+ years and the differences in DFLE between urban and rural areas were statistically significant only at younger ages (until 15 years with marginal level of significance).

**Table 3:** Urban-rural differences in life expectancy, disability-free life expectancy, and proportion of expected life without disability for Bangladeshi female in 2010.

	Life expectancy			Urban		Rural		Proportion of life without disability			
Age	Urban	Rural	Differences in LE^a^ (Urban—Rural)	DFLE	95% CI	DFLE	95% CI	Urban	Rural	Differences in DFLE^b^ (Urban—Rural)	p value^c^
0	69.82	69.26	0.56	58.08	57.41–58.74	56.62	56.14–57.10	83.18	81.74	1.46	0.02
1	71.11	70.70	0.41	59.00	58.31–59.68	57.62	57.13–58.12	82.96	81.51	1.37	0.05
5	67.55	67.35	0.21	55.41	54.72–56.10	54.21	53.71–54.71	82.03	80.50	1.20	0.05
10	62.77	62.73	0.04	50.66	49.96–51.35	49.60	49.10–50.10	80.70	79.06	1.06	0.10
15	58.02	58.02	0.00	46.00	45.30–46.69	44.93	44.43–45.43	79.27	77.44	1.07	0.10
20	53.21	53.35	-0.15	41.24	40.54–41.93	40.33	39.83–40.83	77.50	75.59	0.90	0.20
25	48.46	48.75	-0.29	36.54	35.84–37.23	35.75	35.24–36.25	75.40	73.34	0.79	0.20
30	43.67	44.11	-0.44	31.88	31.18–32.57	31.28	30.77–31.79	72.99	70.91	0.60	>0.20
35	38.80	39.34	-0.54	27.35	26.66–28.04	26.79	26.29–27.30	70.49	68.09	0.56	>0.20
40	34.10	34.69	-0.59	23.10	22.42–23.79	22.57	22.07–23.07	67.76	65.06	0.54	>0.20
45	29.54	30.14	-0.60	19.18	18.50–19.87	18.64	18.15–19.14	64.94	61.85	0.54	>0.20
50	25.12	25.69	-0.57	15.56	14.88–16.23	14.97	14.48–15.46	61.93	58.26	0.59	>0.20
55	21.04	21.95	-0.92	12.43	11.77–13.09	12.08	11.60–12.56	59.09	55.03	0.35	>0.20
60	17.58	18.11	-0.54	9.63	8.97–10.29	9.09	8.62–9.57	54.79	50.19	0.54	>0.20
65	14.20	14.89	-0.69	7.10	6.45–7.75	6.73	6.25–7.21	50.01	45.19	0.37	>0.20
70	11.18	11.95	-0.77	4.93	4.29–5.56	4.69	4.21–5.16	44.08	39.23	0.24	>0.20
75	8.64	9.34	-0.70	3.15	2.51–3.78	3.37	2.88–3.86	36.42	36.08	-0.22	>0.20
80+	7.80	7.84	-0.04	2.48	1.74–3.23	2.65	2.15–3.16	31.87	33.82	-0.17	>0.20

DFLE = disability-free life expectancy; CI = confidence interval of DFLE

^a^ differences are not statistically tested due to lack of required information

^b^ differences are statistically tested with z-statistic

^c^ p values are level of significance for a two-tailed test for differences in DFLE between urban and rural areas.

Life expectancy at birth was 0.56 years higher in the case of urban females compared to rural females, whereas DFLE at birth was 1.46 years higher in the case of urban males compared to rural males. At age 65, an urban female could expect 0.69 years shorter LE but 0.37 years longer DFLE compared to a rural female. The differences in DFLE between urban and rural areas were higher than the differences in LE at every age except for the last age group (open ended interval of the life table). An urban female could expect a higher proportion of life at each age without disability than could a rural female. At birth, an urban female could expect 83% of her remaining life years without disability, at age 65 one could expect 50% of remaining life years without disability, and age 80+ one could expect only 32% of remaining life years without disability. But a rural female could expect 82% of remaining life years without disability at birth, 45% of life years without disability at age 65, and only 34% of remaining life years without disability at age 80+ years.

## Discussion

A higher rate of age-sex-specific mortality was observed in rural areas compared to urban areas with a few exceptions. A statistically insignificant but higher rate of age-sex-specific disability was also observed in rural areas compared to urban areas with a few exceptions. This study revealed significant differences in DFLE from birth to age 15 for both sexes between urban and rural areas in Bangladesh. At all ages, urban males had longer LE, longer DFLE and shorter LE with disability in both numbers and proportions compared to rural males except for age 75. At age 20+ years, rural females had longer LE than urban females; however at all ages, urban females had longer DFLE and shorter LE with disability in both numbers and proportions than rural females except for age 75+. The differences in DFLE exceeded the differences in LE for both sexes at almost all ages. A noticeably lower percentage of remaining life years without disability were expected by older people than younger people regardless of sex in all areas with marginal differences between urban and rural areas across the ages.

The pattern of higher mortality regime might be attributed to less maternal and child health care services, less general health care services in rural areas than urban areas. Lower education induces lower income opportunities for rural people compared to urban people and consequently, rural people have less ability to afford modern health care services than urban people. Health care services and socio-economic status could also be the causes of higher mortality rates in rural areas than in urban areas in Bangladesh and might also be the causes of higher disability rates among rural people than urban people. More in-depth analysis is required for explaining this phenomenon. The most socially disadvantaged people were reported to have the highest rate of disability in several studies [32, 36] while the disability rate was reported to be higher among rural people and people from a lower socio-economic status than their counterparts in Bangladesh [25]. Higher mortality and disability rates among rural people in Bangladesh call for interventions that could reduce both these rates.

The findings of shorter LE and shorter DFLE for rural people than urban people is in line with other studies based on regional, ethnic and socio-economic variations in health expectancy in different settings [12, 37–40], that is, disadvantaged people and people with a low level of education expected shorter LE and shorter DFLE than their counterparts. Rural people were found to have higher mortality and disability rates than urban people that made urban people have higher LE as well as higher DFLE in terms of numbers and proportions. The differences in DFLE exceeded the differences in LE for both sexes. It suggests that urgent action should be taken to reduce inequalities in DFLE between urban and rural areas. Reducing mortality and disability rates by providing necessary health services to all, particularly in rural areas, and by creating income opportunities and removing regional inequalities would also help achieve equality in DFLE between urban and rural areas.

In line with other studies [13, 17, 21], older people had a shorter proportion of remaining life without disability than younger people in the current study. Women were found to have shorter proportion of life without disability compared to men in both urban and rural areas. Attention should thus be given to elderly people, in particular to older women, when reducing inequalities in DFLE between urban and rural areas.

The self-reported data on disability was used in this study that could be a possible source of bias but self-reported data on functional disability was reported to be consistent with medical diagnoses [41]. Although the HIES 2010 administered the Washington Group’s short set of six disability-related questions to all ages to report disability prevalence, it could accommodate two different modules of disability-related questions by Washington Group for children aged below 5 years and for 5–17 years for better reporting of disability prevalence at age 17 years and under. Some elderly people might have been unable to respond to the interviewers, and some proxy respondents might have been interviewed. However, the HIES 2010 mentioned nothing about proxy respondents. The HIES 2010 interviewed non-institutionalised populations, and life tables were computed based on the mortality information of all non-institutionalised (i.e. community people) and institutionalised population. The DFLE might thus be over-estimated if institutionalised populations had higher disability rates than people in the community, or the DFLE might be under-estimated if the institutionalised population had lower disability than people in the community. The same disability rate was assumed for both the community and institutionalised populations in this study. We were unable to accommodate the sampling weights to account for the complex sampling design as the HIES 2010 did not provide any weights for the individuals which could be a possible source of bias for the representativeness of the sample by urban-rural areas. Despite these limitations, the most reliable and relevant nationally representative data with a large number of samples delineated a clear scenario of urban-rural differences in DFLE for people living in Bangladesh.

## Conclusion

This study provides the first estimations of DFLE by urban-rural areas with its differentials for Bangladesh. The findings have important implications for helping to reduce urban-rural inequalities in health expectations. The urban-rural differences in economic development, health care facilities and access, educational opportunities and infrastructures are recommended for reduction in order to help achieve urban-rural equality in DFLE. Socio-economic conditions including infrastructure development, accessible and affordable health care services for rural people should receive due attention in health policy making in Bangladesh.
